# Integrating differential privacy into federated multi-task learning algorithms in **dsMTL**

**DOI:** 10.1093/bioadv/vbaf298

**Published:** 2025-11-23

**Authors:** Roman Schefzik, Han Cao, Sivanesan Rajan, Xavier Escribà-Montagut, Juan R González, Emanuel Schwarz

**Affiliations:** Hector Institute for Artificial Intelligence in Psychiatry, Central Institute of Mental Health, Medical Faculty Mannheim, Heidelberg University, 68159 Mannheim, Germany; Department of Psychiatry and Psychotherapy, Central Institute of Mental Health, Medical Faculty Mannheim, Heidelberg University, 68159 Mannheim, Germany; Department for Theoretical Neuroscience, Central Institute of Mental Health, Medical Faculty Mannheim, Heidelberg University, 68159 Mannheim, Germany; Hector Institute for Artificial Intelligence in Psychiatry, Central Institute of Mental Health, Medical Faculty Mannheim, Heidelberg University, 68159 Mannheim, Germany; Department of Psychiatry and Psychotherapy, Central Institute of Mental Health, Medical Faculty Mannheim, Heidelberg University, 68159 Mannheim, Germany; Barcelona Institute for Global Health (ISGlobal), 08003 Barcelona, Spain; Barcelona Institute for Global Health (ISGlobal), 08003 Barcelona, Spain; Hector Institute for Artificial Intelligence in Psychiatry, Central Institute of Mental Health, Medical Faculty Mannheim, Heidelberg University, 68159 Mannheim, Germany; Department of Psychiatry and Psychotherapy, Central Institute of Mental Health, Medical Faculty Mannheim, Heidelberg University, 68159 Mannheim, Germany

## Abstract

**Motivation:**

Multi-task learning (MTL) enables simultaneous learning of related regression or classification tasks by exploiting shared information. The R package dsMTL provides a computational framework for federated MTL approaches, supporting the analysis of sensitive, individual-level data from geographically distributed data sources using the DataSHIELD platform. While the current architecture provides comprehensive data security mechanisms, these are not specifically tailored to MTL models. In particular, these models may still be vulnerable to membership inference attacks, attempting to determine whether a specific individual was included in a given training set using the model.

**Results:**

To further enhance the privacy-preserving capabilities of dsMTL and protect against such attacks, differential privacy using the Laplace mechanism is integrated into dsMTL as a novel optional feature. This approach aims to obscure individual-level characteristics from the model while retaining group-level differences. The differential privacy implementation is validated in both simulation studies and a case study identifying schizophrenia patients from gene expression data. For practical utility, it is crucial to find an adequate balance between the degree of privacy protection and the conservation of model performance by choosing a reasonable privacy parameter within the differential privacy mechanism.

**Availability and implementation:**

dsMTL is open-source and available at https://github.com/transbioZI/dsMTLBase (server-side) and https://github.com/transbioZI/dsMTLClient (client-side).

## 1 Introduction

Multi-task learning (MTL) (Caruana 1997, Zhang and Yang 2018) aims at simultaneously learning multiple related regression or classification tasks, thereby exploiting information between them. It has been applied to a vast range of research areas, including bioinformatics (Zhang and Yang 2018).

In particular in the (bio)medical and health sciences, data is typically not freely exchangeable across institutions, as the privacy of individuals has to be protected. To address this issue, a prominent remedy is to rely on federated learning strategies (Brauneck *et al.* 2023), where algorithms are simultaneously learned at different, geographically distributed institutions and optimized through a privacy-preserving exchange of parameters, thus pursuing the strategy of bringing the algorithm to the data and not vice versa.

The R (R Core Team 2024) package dsMTL (Cao *et al.* 2022) offers a computational framework for federated MTL. It has been developed on the basis of the DataSHIELD (ds) platform (Wilson *et al.* 2017, Marcon *et al.* 2021), providing functionality regarding data management, transmission and security, where data are analyzed behind a given institution’s firewall, and only algorithm parameters not disclosing personally identifiable information are exchanged (Cao *et al.* 2022).

While DataSHIELD provides broad security mechanisms, these are not specific to machine learning applications, and DataSHIELD relies on the prevalent assumption that summary statistics are safe to share. However, machine learning models may be vulnerable to data inference attacks, and thus, there is a potential for machine learning-specific privacy leaks. In particular, membership inference attacks (MIAs) (Shokri *et al.* 2017, Hu *et al.* 2022) attempt to decide whether an individual was included in a given training set using the model. As such attacks require a complete model for inference, a protective measure may e.g. be to return an incomplete model only. Alternatively, a protection against MIAs can be obtained by applying differential privacy (Dwork 2006, Dwork and Roth 2014). This concept is aimed at erasing individual-level characteristics from the model while retaining group differences and has already been successfully implemented for a variety of models (Shokri *et al.* 2017), in particular for analyzing omics data in federated settings (Escribà-Montagut *et al.* 2024).

**Figure 1.**
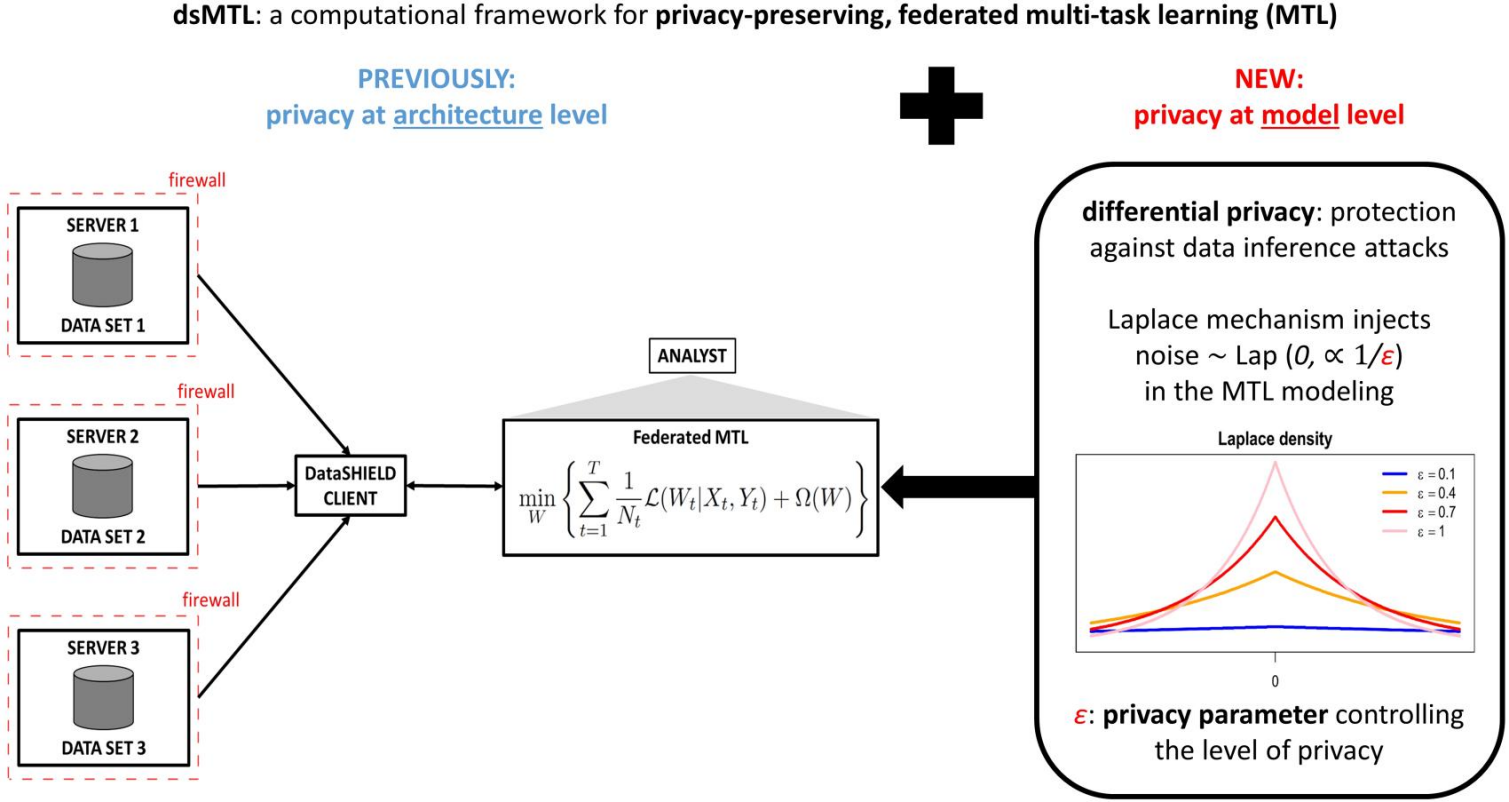
Illustration of dsMTL, in the spirit of Figure 1 in Cao *et al.* (2022). For federated MTL, multiple datasets are stored at different institutions. The previous implementation of dsMTL (left) is built on the DataSHIELD platform, where data are analyzed behind a given institution’s firewall and only algorithm parameters, but not personally identifiable information, are exchanged during the analysis. While this setup provides privacy protection at an *architecture* level, we on top integrate differential privacy into dsMTL (right), to additionally offer protection against MIAs at a statistical *model* level. Differential privacy is achieved using the Laplace mechanism and relies on a suitable choice of a privacy parameter ε>0.

Specifically, the concept of differential privacy can be integrated into MTL approaches (Hu *et al.* 2021), and in particular into federated MTL frameworks (Okegbile *et al.* 2023, Benouis *et al.* 2024), to further strengthen their privacy-preserving nature by offering a protection against MIAs. In our work here, we add differential privacy components to the MTL approaches implemented in the existing dsMTL framework for federated MTL, using the common Laplace (noise) mechanism to achieve differential privacy. Thus, we extend the dsMTL framework by including an additional privacy component, which may optionally be applied according to user needs (Fig. 1).

## 2 Methods

### 2.1 Multi-task learning algorithms in dsMTL

We here focus on three different MTL implementations for regression and classification, respectively, namely dsMTL_L21, dsMTL_Trace and dsMTL_Net (Cao *et al.* 2022). These MTL methods follow a consistent formulation and aim at minimizing the same objective:


$$\underset{W}{\min}\left\{\sum_{t=1}^{T}\frac{1}{N_{t}}L(W_{t}|X_{t},Y_{t})+\Omega (W)\right\}.$$

Here, $X_{t}\in R^{N_{t}\times P}$ denotes the predictor matrix and $Y_{t}\in R^{N_{t}\times 1}$ the corresponding response vector for task t∈{1,…,T}, with *T* referring to the number of tasks, $N_{t}$ to the number of subjects for task *t* and *P* to the number of predictors (where all tasks share the same predictor space). Moreover, L is a loss function, where least square loss is used for regression and logistic loss for classification, respectively. The output of the MTL estimation is a coefficient matrix $W:=(W_{1},\ldots ,W_{t},\ldots ,W_{T})\in R^{P\times T}$ for all tasks, with $W_{t}$ being the *t*-th column of *W*, consisting of the coefficient vector of length *P* corresponding to task *t*. Thus, *W* consists of entries $w_{p,t}$ referring to predictor p∈{1,…,P} and task t∈{1,…,T}. Lastly, Ω(W) is a regularization term that prevents overfitting and incorporates prior information and knowledge transfer among tasks. Specifically,


Ω(W):=λN(W)+CS(W),

where *N* is a non-smooth function for creating sparsity and *S* is a smooth function with the ability to stabilize the solution, with λ≥0 and C≥0 being hyperparameters to control the strengths of these penalty terms.

Our different considered MTL methods can be distinguished based on the specific choice of *N* and *S*, respectively:

- dsMTL_L21: Here, $N(W):=||W|{|}_{2,1}$ and $S(W):=||W|{|}_{F}^{2}$, with $||\cdot |{|}_{2,1}$ and $||\cdot |{|}_{F}$ denoting the L21 norm and the Frobenius norm, respectively. This MTL variant allows for cross-task regularization and joint feature selection across tasks, building on the well-known lasso method (Tibshirani 1996), in order to identify outcome-associated signatures with a reduced number of features shared across tasks.
- dsMTL_Trace: Here, $N(W):=||W|{|}_{*}$ and $S(W):=||W|{|}_{F}^{2}$, with $||\cdot |{|}_{*}$ denoting the trace norm. This MTL method constrains the coefficient vectors in a shared low-dimensional space during the training procedure, while retaining a sufficient amount of variability for each coefficient vector to learn a given pattern, resulting in improved generalizability of the models.
- dsMTL_Net: Here, $N(W):=||W|{|}_{1}$ and $S(W):=||GW|{|}_{F}^{2}$, with $||\cdot |{|}_{1}$ denoting the L1 norm, and *G* a specifically designed matrix describing a network structure. This MTL approach explicitly models task-task relationships and incorporates a task-task network as the shared structure in order to improve biological interpretability.

We refer to (in particular the supplement in) Cao *et al.* (2022) for more information on the methodologies and implementation details.

### 2.2 Differential privacy

The notion of differential privacy (Dwork 2006, Dwork and Roth 2014) is based on guarantees that a randomized algorithm behaves similarly on similar input datasets that differ in one data point. Here, we specifically use the concept of ε-differential privacy. For a formal description, let $D_{1},D_{2}\in D$ be two datasets that are obtained from one another by removing one data point, denoted by $D_{1}∼D_{2}$, and let *A* denote a randomized algorithm that produces an output in a space O on input data in D. Then, *A* is ε-differentially private if


$$P[A(D_{1})=S]\leq \text{exp}(\varepsilon )\times P[A(D_{2})=S]$$

for all datasets $D_{1},D_{2}\in D$, $D_{1}∼D_{2}$, differing in at most one data point, and all subsets of outcomes S⊆O, with ε>0 being the privacy parameter defining the level of privacy.

For the specific implementation of differential privacy, we here rely on the Laplace mechanism, a commonly used approach to achieve differential privacy, see Section 3.3 in Dwork and Roth (2014). This strategy adds noise to the output of a function $f:D\to R^{d}$, where the noise is drawn from a Laplace distribution Lap(0,Δf/ε) with location parameter 0 and scale parameter Δf/ε. Precisely, the Laplace mechanism is defined as $f(D)+(Z_{1},\ldots ,Z_{d})$ with random variables $Z_{i}{∼}^{\mathrm{iid}}\text{Lap}(0,\Delta f/\varepsilon )$ for i∈{1,…,d} and D∈D. Here, Δf is the $\ell_{1}$ sensitivity given by


$$\Delta f=\underset{D_{1}∼D_{2}}{\max}\{||f(D_{1})-f(D_{2})|{|}_{1}\},$$

where the maximum is taken over all pairs of datasets $D_{1},D_{2}\in D$, $D_{1}∼D_{2}$, differing in at most one data point, and $||.|{|}_{1}$ denotes the $\ell_{1}$-norm. In particular, the Laplace mechanism theoretically requires a bound on the $\ell_{1}$ sensitivity Δf. However, typically, and in particular for more involved functions *f*, Δf cannot easily be bounded analytically. In such cases, a sensitivity sampling method following Rubinstein and Aldà (2017) can be used to assess Δf practically. Specifically, starting with an original training dataset $D_{1}$, another set $D_{2}$ is created by removing exactly one randomly chosen data point from $D_{1}$. Then, a value *l* is calculated via $l:=||f(D_{1})-f(D_{2})|{|}_{1}$. By conducting this procedure in total *M* (for instance, M:=100) times, *M* values $l_{1},\ldots ,l_{M}$ are obtained, and Δf is finally computed via $\Delta f:=\max\{l_{1},\ldots ,l_{M}\}$.

The choice of ε is crucial in the differential privacy implementation. It is typically recommended to aim for a value of ε≤1 to have a reasonable degree of privacy, and in many applications, ε is actually chosen between 0.01 and 10 (Lee and Clifton 2011, Hsu *et al.* 2014). However, the specific choice of ε is finally up to the user and may strongly depend on the application at hand. Overall, the smaller ε, the more noise is added and the more privacy there is. Indeed, letting ε→0 enhances the noise, as then, for the scale parameter, Δf/ε→∞ holds. While stronger privacy guarantees are provided by choosing a smaller ε, the corresponding output may be less useful in terms of model performance. Intuitively, setting ε:=∞ corresponds to omitting differential privacy by adding no Laplace noise, as for ε→∞, it holds that Δf/ε→0 for the scale parameter.

### 2.3 Implementation of differential privacy in dsMTL

The dsMTL implementation for federated MTL is split into two R packages: one at the server-side (dsMTLBase) and one at the client-side (dsMTLClient). Basically, the actual MTL algorithms run at the client-side in an iterative process, but the calculations of involved gradients and values of the respective loss functions, which are performed using the protected raw data, happen at the server-side, for each task separately. Here, only estimated model coefficients and values are exchanged and given back to the client-side, but not the raw data, which remain protected at the respective data hosts (Cao *et al.* 2022).

To additionally integrate differential privacy into this federated framework, we add noise obtained by the Laplace mechanism to both (i) the gradients and (ii) the values of the respective loss functions calculated in each iterative run. This basically happens at the client-side. However, in the process of generating the Laplace noise, we need to assess the sensitivities $\Delta f_{1}$ and $\Delta f_{2}$ of the functions $f_{1}$ and $f_{2}$ to compute (i) the gradients and (ii) the values of the respective loss functions, respectively. In each case, this is done using the sensitivity sampling method based on *M* simulation runs by Rubinstein and Aldà (2017). Here, the sensitivity sampling requires the repeated calculations of differences of gradients and values, respectively, of corresponding loss functions based on the original data and the original data with one data point removed, for each task separately. To keep the confidentiality of the raw data, these computations are consequently conducted at the server-side.

Overall, differential privacy is integrated in a modular fashion into dsMTL and can optionally be enabled by the user by specifying two additional arguments compared to the original implementation: the privacy parameter ε and the number of simulation runs *M* for the assessment of the function sensitivity involved in the Laplace mechanisms.

### 2.4 Evaluation procedures and measures

In both simulations and a real-data application, we prove the validity of our differential privacy implementation and in particular evaluate both the robustness against MIAs and the conservation of model performance. To this end, we in particular assess the effect of the choice of the privacy parameter ε>0 in comparison to the non-private implementation not incorporating differential privacy (ε=∞).

#### 2.4.1 Protection against membership inference attacks (MIAs)

On the one hand, we aim at showing the robustness of our differential privacy implementation against MIAs. To this end, we do not use more advanced MIAs for instance based on shadow models (Shokri *et al.* 2017) for our purposes here, but instead consider a comparably simple MIA approach based on comparisons to the average training loss that has been employed previously (Yeom *et al.* 2018, Schamoni *et al.* 2022). Specifically, the attacker is assumed to have access to the average training loss, which may for instance be known due to a security breach or might be estimated if the attacker has some knowledge about the original distribution of the training data. In order to infer the membership of a data point, the attacker applies the model to the data and obtains the respective prediction. Subsequently, the attacker computes the error (loss) on this example by comparing the prediction to the gold label. Eventually, the example is considered to have been part of the training data if the respective computed loss is smaller than the average training loss (Schamoni *et al.* 2022). For our purposes here, we employ the mean squared error (MSE) as the underlying loss function for regression tasks, and the logistic loss accordingly for classification tasks. To evaluate the protection against this MIA provided by our differential privacy implementations, as in Schamoni *et al.* (2022), we here consider the privacy leakage (PL), which is also known as the attacker’s advantage (Yeom *et al.* 2018, Jayaraman and Evans 2019), for different privacy parameters ε. The PL is given by the difference between the true positive rate (TPR) and the false positive rate (FPR) of an MIA:


PL:=TPR−FPR.

Here, for each considered privacy parameter ε, the FPR is computed as the ratio of false positives from an unseen test dataset (“negatives”), and the TPR as the ratio of true positives from the training dataset (“positives”). In particular, to account for the federated setting, the PL is here first calculated for each task (corresponding to one server) separately, but finally the average over the PL values across all considered tasks is used for an overall evaluation.

#### 2.4.2 Conservation of model performance

On the other hand, we assess the effect of the choice of the privacy parameter ε on the MTL algorithm performance results in comparison to the results obtained using the non-private implementation. To this end, for regression, we consider the per cent increase in the mean squared error (MSE), IncMSE, when comparing the ${\text{MSE}}_{P}$ of a private model to the ${\text{MSE}}_{\text{NP}}$ of the non-private model, each averaged over all tasks:


$$\text{IncMSE}:=\frac{{\text{MSE}}_{P}-{\text{MSE}}_{\text{NP}}}{{\text{MSE}}_{\text{NP}}}\times 100\%.$$

Analogously, for classification, we consider an accuracy loss ${\text{AccLoss}}_{\text{AUROC}}$ (Jayaraman and Evans 2019) with respect to area under receiver operating characteristic curve (AUROC), an evaluation strategy that has previously been used by Schamoni *et al.* (2022):


$${\text{AccLoss}}_{\text{AUROC}}:=1-\frac{(2\times {\text{AUROC}}_{P})-1}{(2\times {\text{AUROC}}_{\text{NP}})-1}.$$

Here, ${\text{AUROC}}_{P}$ and ${\text{AUROC}}_{\text{NP}}$ denote the AUROC values corresponding to the considered private and non-private MTL models, respectively, averaged over the tasks. If ${\text{AUROC}}_{\text{NP}}=0.5$, then we set ${\text{AUROC}}_{\text{NP}}=0.5+\eta$ with a random number η drawn from a normal distribution with mean 0.5 and standard deviation $10^{-6}$ to avoid undefined expressions.

## 3 Results

To evaluate the effect of the implementation of differential privacy using the Laplace mechanism on the protection against MIAs and the conservation of MTL model performance, we both consider simulation studies and a real-data case study.

### 3.1 Simulation studies

#### 3.1.1 Settings

We consider each of the implemented MTL approaches (i.e. dsMTL_L21, dsMTL_Trace and dsMTL_Net) in a regression and a classification setting, respectively. While we exemplarily focus on the dsMTL_L21 approach for regression here, detailed simulation settings and results for the other MTL approaches are deferred to the Sections S1.1 and S1.2, available as supplementary data at *Bioinformatics Advances* online.

For each setting, we generate one simulated underlying training and test dataset, respectively, using slightly modified versions of the simulation procedure based on standard normal distribution models previously described in the supplement of Cao *et al.* (2019). First, we compute one non-private model (corresponding to ε=∞), i.e. we take the respective default option in dsMTL and do not specify a privacy parameter ε. In a second step, we apply the differential privacy mechanism by specifying different privacy parameter values ε>0, where we cover a broad spectrum of possible values for ε ranging from 0.001 to 1000. In particular, to assess the function sensitivity required for the differential privacy mechanism, we use the sampling method following Rubinstein and Aldà (2017) based on M:=100 simulation runs here. To account for sampling variability when using the Laplace mechanism for differential privacy, we conduct the model calculation for the private models in total R:=100 times and consider the corresponding distributions of the evaluation measures from Section 2.4 for the assessment of the differential privacy implementations. Throughout all examples, concerning the control of the respective MTL optimization procedure, we use the 0 matrix as starting point, a maximum number of iterations of 50, and a tolerance of the acceptable precision of solution to terminate the algorithm of 0.01. Moreover, the termination rule to determine whether the optimization converges here considers the last two objective values and checks whether the corresponding decrement was close enough to zero.

Specifically for dsMTL_L21 for regression (implementing a consistent selection of predictors across the two tasks), we consider P:=1000 predictors, T:=2 tasks and $N_{1}=N_{2}=500$ observations, and training and test data, respectively, is generated as follows. For each task t∈{1,2}, we create a (500×1000) predictor matrix $X_{t}$ with entries drawn from a standard normal distribution, as well as a (1000×1) coefficient vector $W_{t}$ with entries also drawn from a standard normal distribution, aggregated in a coefficient matrix $W:=(W_{1},W_{2})\in R^{1000\times 2}$. Then, for 50% of the predictors (i.e. 50% of the rows of *W*), the corresponding entries are set to exactly zero for both tasks (i.e. columns of *W*). For each task t∈{1,2}, a response vector $Y_{t}\in R^{500\times 1}$ is then derived via $Y_{t}:=X_{t}W_{t}+0.5\delta$, where the (500×1) vector δ consists of entries drawn from a standard normal distribution. For the MTL model calculation in both the non-private and the private cases, we here set the model hyperparameters to λ:=1.2 and C:=1, respectively.

#### 3.1.2 Results

Regarding the example for dsMTL_L21 in case of regression, the smaller ε in the private models the more Laplace noise is injected into the non-private model and thus the more the estimated model coefficients deviate from those obtained by the non-private model (Section S1.2.1 and Fig. S1, available as supplementary data at *Bioinformatics Advances* online). For large values of ε, the estimated model coefficients of the respective private models strongly resemble those estimated by the non-private model.

**Figure 2.**
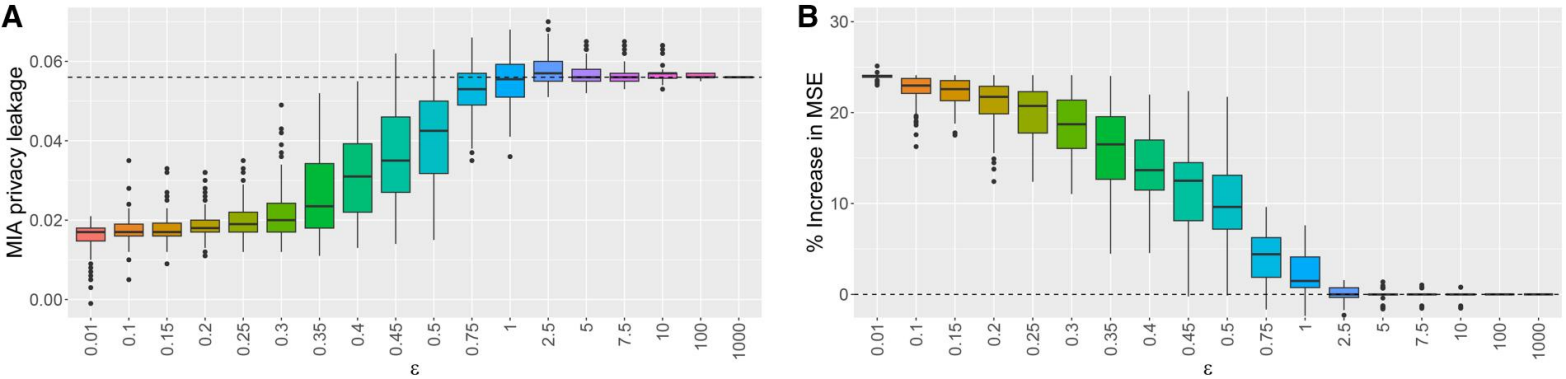
Differential privacy in the simulation study for dsMTL_L21 for regression: for different values of the privacy parameter ε>0, boxplots showing the distributions over 100 runs of (A) privacy leakages against an MIA of the private models, where the horizontal dashed line indicates the MIA privacy leakage of the non-private model; (B) percentage increases in MSE of the private models compared to the non-private model, where the horizontal dashed line at zero indicates an identical performance in terms of the MSE for the non-private and the private models.

Here, the non-private model appears to have already a rather good protection against MIAs, as mirrored by the corresponding comparably small PL value. Compared to the non-private model, the great majority of the private models appears to reduce the PL again, even though effects may typically be rather small (Fig. 2A).

The non-private model has an average (median) MSE of 400.47 over the two tasks. The smaller ε the more predictive skill is lost (expressed by an increase of the MSE) in the private models. For large values of ε, the model performance from the non-private model is virtually completely retained (Fig. 2B).

Taking the (non-)selected predictors by the non-private model as references, there are typically far more predictors wrongly selected by the private models than missed by the private models. While the respective false negative rates are low across all values of ε, the corresponding false positive rates decrease with increasing ε (Section S1.2.1 and Figs S2 and S3, available as supplementary data at *Bioinformatics Advances* online).

**Figure 3.**
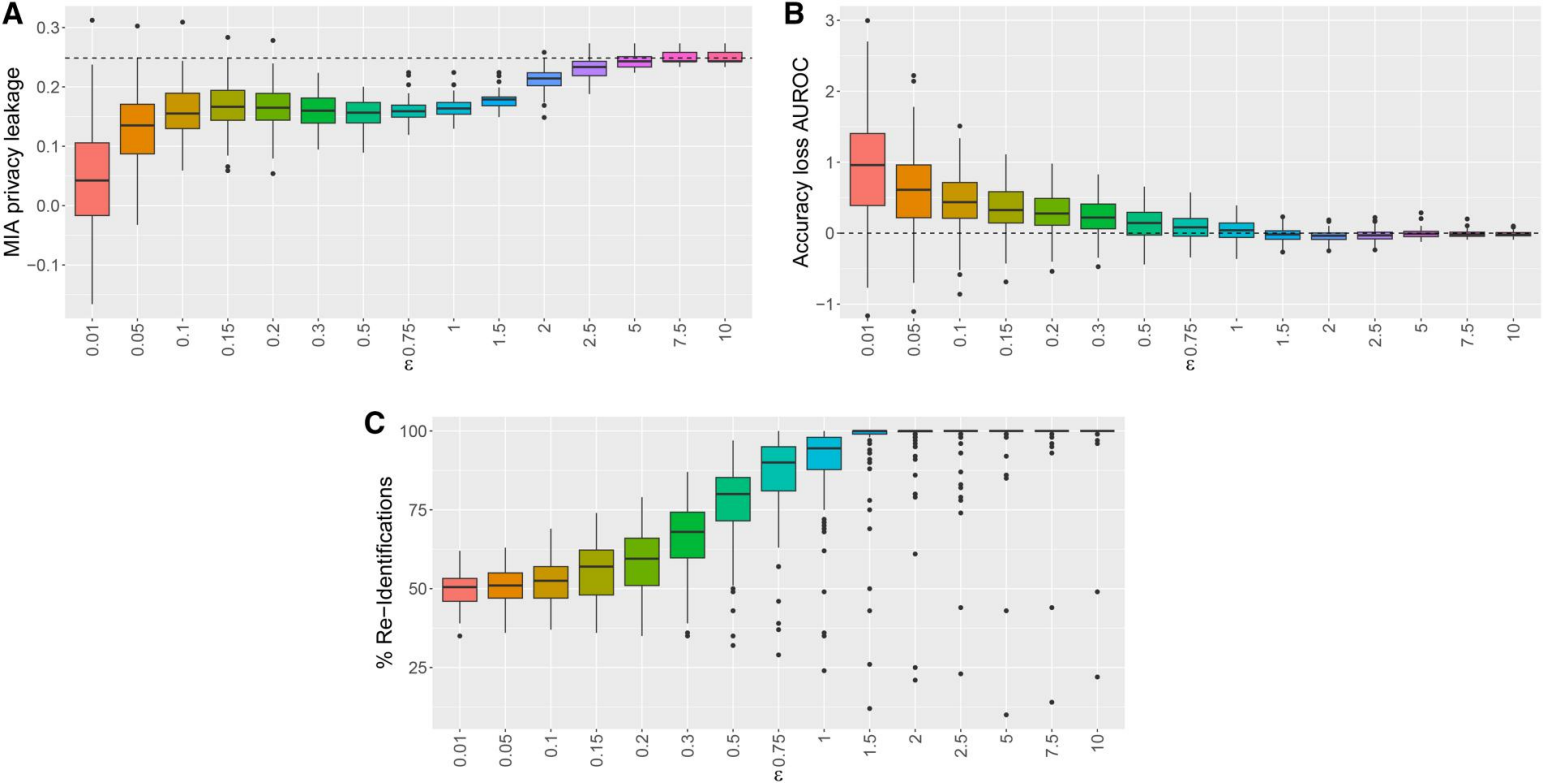
Differential privacy in the case study: for different values of the privacy parameter ε>0, boxplots showing the distributions over 100 runs of (A) privacy leakages against an MIA of the private models, where the horizontal dashed line indicates the MIA privacy leakage of the non-private model; (B) accuracy losses of the private models compared to the non-private model with respect to the AUROC, where the horizontal dashed line at zero indicates an identical performance in terms of the AUROC for the non-private and the private models; (C) percentages of re-identified genes from the set G by the private models, where G refers to the top 100 genes with the highest average model coefficients derived from those genes that are identified by the non-private model to have non-zero model coefficients with the same sign across all tasks.

In our example here, a reasonable trade-off between protection against MIAs and conservation of model performance may be realized best for private models based on values of ε in the range of around 0.4 to 0.75.

Similar deductions can be made for the other considered MTL implementations (Sections S1.2.2–S1.2.6 and Figs S4–S13, available as supplementary data at *Bioinformatics Advances* online). Overall, compared to the non-private model, private models based on smaller values of ε offer an increased protection against MIAs, but at the cost of a potentially considerable loss of model performance. Vice versa, private models based on larger values of ε virtually fail to provide an additional protection against MIAs, but retain the predictive performance of the non-private model. Suitable ranges of ε values that balance privacy protection and preservation of model performance can be determined for each setting; however, these ranges vary depending on factors such as the considered MTL approach and the overall task (regression versus classification).

### 3.2 Case study

#### 3.2.1 Setting

As a real-data case study, we here specifically consider an application of the dsMTL_L21 approach to the binary case-control classification task of identifying individuals with schizophrenia (SCZ) (McCutcheon *et al.* 2020) based on gene expression data.

SCZ is a severe mental disease which is manifested by the combined occurrence of psychotic symptoms and cognitive malfunctions. It affects around 1% of the global population and leads to immense societal and economic burdens (Chong *et al.* 2016, Velligan and Rao 2023).

Specifically, we here consider three independent brain cortical microarray gene expression datasets relating to schizophrenia case-control (SCZ/Ctrl) cohorts publicly available from the Gene Expression Omnibus (GEO) repository: the GEO dataset GSE21138 (Narayan *et al.* 2008, Tang *et al.* 2011, 2012), the GEO dataset GSE35977 (Chen *et al.* 2013, 2018), and the GEO dataset GSE53987 (Lanz *et al.* 2019). First, these datasets are synchronized and pre-processed, including steps such as outlier removal, adjustment of confounders and *z*-standardization, where a detailed description of the pre-processing procedure is outlined (in the supplement) in Cao *et al.* (2024). After the pre-processing, GSE21138 comprises $N_{1}:=52$ (27 SCZ; 25 Ctrl) individuals, GSE35977 comprises $N_{2}:=100$ (51 SCZ; 49 Ctrl) individuals, and GSE53987 comprises $N_{3}:=33$ (15 SCZ; 18 Ctrl) individuals, where each dataset consists of the same P:=17 166 genes as predictors.

In our case study, we use the GSE21138 and GSE35977 datasets for training. That is, each of these datasets is considered as one of T:=2 tasks, where tasks correspond to different cohorts, comprising different individuals, here. The GSE53987 dataset is used for testing.

First, we compute one non-private dsMTL_L21 model (corresponding to ε=∞). Then, we apply the differential privacy mechanism by considering a broad spectrum of different privacy parameter values ε>0. To account for sampling variability when using the Laplace mechanism for differential privacy, we conduct the model calculation for the private models for each value of ε in total R:=100 times and consider the corresponding distributions of the evaluation measures from Section 2.4 and measures for the assessment of the differential privacy implementations.

For the dsMTL_L21 model calculation in both the non-private and the private cases, we here set the model hyperparameters to λ:=0.15 and C:=1, respectively, where the sensitivity sampling required for the calculations in the respective differentially private settings is based on M:=100 runs here. Regarding the control of the dsMTL_L21 optimization procedure, we here use the 0 matrix as starting point, a maximum number of iterations of 50, and a tolerance of the acceptable precision of solution to terminate the algorithm of 0.01. Moreover, the termination rule to determine whether the optimization converges here considers the last two objective values and checks whether the corresponding decrement was close enough to zero.

#### 3.2.2 Results

The private models based on the small values of ε∈{0.01,0.05} reduce the PL most compared to the non-private model (Fig. 3A). However, a notable reduction of the PL, and thus an are effective protection against MIAs, is also achieved for ε∈{0.1,0.15,0.2,0.3,0.5,0.75,1,1.5,2}. In contrast, the private models based on ε∈{2.5,5,7.5,10} basically exhibit the same PL as the non-private model and thus fail to provide an additional protection against MIAs.

The non-private model has an average (median) AUROC of 0.613 over the two tasks (training cohorts). The smaller ε the more predictive skill in terms of AUROC is lost in the private models. In contrast, for larger values of ε, the model performance from the non-private model is virtually completely retained (Fig. 3B).

In the specific application here, we also consider to what extent genes that have been selected as important predictors by the non-private model are re-identified by the respective private models. The non-private model identifies 559 genes as predictors with non-zero coefficients over both tasks. Of these, 224 have a same model coefficient sign (positive or negative) over both tasks. We here order these 224 genes according to their respective average model coefficients and take the top 100 genes with the highest average coefficients as a reference gene set G. Among others, G includes genes that have already been shown to play an important role in the pathophysiology of SCZ, such as NPY (Itokawa *et al.* 2003, Eaton *et al.* 2007) and PIAS3 (Rubio *et al.* 2013). For each private model $m_{P}$, i.e. for each ε and each of the respective R=100 runs, we similarly compute a set of genes $G_{m_{P}}$ that are selected as predictors across both tasks and exhibit the same model coefficient sign. Then, for each of the 100 genes in G, we check whether it is present in $G_{m_{P}}$, i.e. whether it is re-identified by the corresponding private model. The number of the re-identified genes from G then obviously corresponds to the percentage of the re-identified genes from G and may be seen as a measure for the conservation of biological interpretability by the private model. Summarizing these checks (Fig. 3C), for smaller values of ε from 0.01 to around 0.2, the percentage of re-identified genes is comparably low. In contrast, for larger values of ε from around 1.5 to 10, the genes are virtually completely re-identified. For medium values of ε from around 0.3 to 1, the percentage of re-identified genes is at a satisfying to good level. These results overall appear to be well in accordance with those regarding the accuracy loss in terms of AUROC (Fig. 3B).

Overall, a reasonable trade-off between protection against MIAs and conservation of model performance may be realized best for private models based on values of ε in the range of around 0.3 to 1 here. Such an adequate choice of ε also ensures that the private model retains biological interpretability, as evidenced by the re-identification of genes crucial to the pathophysiology of SCZ.

## 4 Discussion

We here integrated differential privacy components based on the Laplace mechanism as a valuable additional feature into the R package dsMTL for federated MTL, in order to circumvent MTL leak and vulnerability to e.g. MIAs (Yan *et al.* 2024), which cannot be fully mitigated by the technical architecture of the DataSHIELD ecosystem alone.

While differential privacy is in particular appealing and sound from a purely theoretical point of view, and despite its popularity, an indiscriminate application may, however, be problematic. As demonstrated by our simulations and case study, a useful practical application strongly relies on a suitable specification of the privacy parameter ε, where it is crucial to find a reasonable trade-off between privacy level and conserved model performance (Subramanian 2023).

As witnessed by the varying levels of MIA privacy leakages in our studies, the vulnerability of non-private models to MIAs, and consequently the potential need for differential privacy, depends heavily on context. In some scenarios, the risk is already quite low, and differential privacy may not be necessary. In others, privacy risks are more pronounced and can be significantly reduced through its application. It is important to note that we here employed only a relatively simple MIA variant to illustrate this vulnerability. While this basic attack already revealed measurable privacy leakage, more advanced attacks, such as those leveraging shadow models (Shokri *et al.* 2017), can be even more effective and thus pose greater threats. This further highlights the value of differential privacy as an additional layer of protection.

While Bambauer *et al.* (2014) harshly criticized differential privacy’s practical utility in general, we do not advocate for or against its universal adoption here. Instead, we present it as an additional optional feature within the federated framework of dsMTL, empowering users to make informed decisions based on their specific needs. Ultimately, whether and to what extent differential privacy should be employed depends on the user’s objectives, risk tolerance, and the context of the application. To support this, we provide an optional differential privacy implementation that enhances data security for users who prioritize it, while still allowing others to focus on optimizing model performance.

While specifically implemented for MTL in federated settings here, differential privacy can also be integrated into MTL algorithms for standard non-federated settings (Section S2.1, available as supplementary data at *Bioinformatics Advances* online).

There are several avenues for future work regarding dsMTL, including the integration of alternative privacy-preserving concepts or the reduction of computation time (Section S2.2, available as supplementary data at *Bioinformatics Advances* online).

## 5 Conclusion

In our work here, differential privacy based on the Laplace mechanism is integrated as an optional feature into the R package dsMTL for federated MTL, in order to further strengthen its privacy-preserving nature by offering an additional protection against data inference attacks. For practical utility, it is crucial to find an appropriate balance between the degree of achieved privacy and conserved model performance.

## Supplementary Material

vbaf298_Supplementary_Data

## Data Availability

The case study was based on raw datasets that are publicly available from the Gene Expression Omnibus (GEO) repository under GEO accessions GSE21138, GSE35977, and GSE53987, respectively.

## Software availability

dsMTL is freely available at https://github.com/transbioZI/dsMTLBase (server-side R package) and https://github.com/transbioZI/dsMTLClient (client-side R package). The code associated with this publication is archived on Zenodo at https://doi.org/10.5281/zenodo.17570010 and https://doi.org/10.5281/zenodo.17569955.
