# Supplementary material for: Integrating differential privacy into federated multi-task learning algorithms in dsMTL
Source: Bioinform Adv. 2025 Nov 23;5(1):vbaf298. doi: 10.1093/bioadv/vbaf298 (PMC12701803; doi:10.1093/bioadv/vbaf298)
Supplement: vbaf298_Supplementary_Data [file vbaf298_supplementary_data.pdf]

Supplementary Material

Integrating differential privacy into federated multi-task learning algorithms in dsMTL

Roman Schefzik, Han Cao, Sivanesan Rajan, Xavier Escribà-Montagut,
Juan R. González and Emanuel Schwarz

Contents

S1 Simulation studies **2**
S1.1 Settings 2
S1.1.1 dsMTL_L21 for regression 2
S1.1.2 dsMTL_L21 for classification 3
S1.1.3 dsMTL_Trace for regression 3
S1.1.4 dsMTL_Trace for classification 3
S1.1.5 dsMTL_Net for regression 4
S1.1.6 dsMTL_Net for classification 4
S1.2 Results 5
S1.2.1 dsMTL_L21 for regression 5
S1.2.2 dsMTL_L21 for classification 8
S1.2.3 dsMTL_Trace for regression 10
S1.2.4 dsMTL_Trace for classification 12
S1.2.5 dsMTL_Net for regression 14
S1.2.6 dsMTL_Net for classification 16

S2 Extended discussion **18**
S2.1 Non-federated versions of differential privacy implementations for MTL 18
S2.2 Outlook and potential future work 18

References **19**

S1 Simulation studies

S1.1 Settings

To demonstrate the effect of the implementation of differential privacy using the Laplace mechanism, we here consider one example for each of the in total six implemented approaches: dsMTL_L21, dsMTL_Trace and dsMTL_Net, respectively, for classification and regression, respectively.
For each setting, we generate one simulated underlying training and test data set, respectively, using slightly modified versions of the simulation procedure based on standard normal distribution models previously described in the supplement of Cao et al. (2019). First, we compute one non-private model (corresponding to $\varepsilon = \infty$), i.e., we take the respective default option in `dsMTL` and do not specify a privacy parameter ε. In a second step, we apply the differential privacy mechanism by specifying different privacy parameter values $\varepsilon > 0$, where we cover a broad spectrum of possible values for ε ranging from 0.001 to 1000. In particular, to assess the function sensitivity required for the differential privacy mechanism, we use the sampling method following Rubinstein and Aldà (2017) based on $M := 100$ simulation runs here. To account for sampling variability when using the Laplace mechanism for differential privacy, we conduct the model calculation for the private models in total 100 times and consider the corresponding distributions of the evaluation measures for the assessment of the differential privacy implementations.
For details on the generation of training and test data sets and the choice of the number of predictors, observations and tasks, respectively, as well as the model hyperparameters in the different settings, see the corresponding specifications below. Note that the specifications regarding the dsMTL_L21 approach for regression are also outlined in the main text, but we repeat them here for the sake of consistency and better comparability.
Throughout all examples, concerning the control of the respective MTL optimization procedure, we use the 0 matrix as starting point, a maximum number of iterations of 50, and a tolerance of the acceptable precision of solution to terminate the algorithm of 0.01. Moreover, the termination rule to determine whether the optimization converges here considers the last two objective values and checks whether the corresponding decrement was close enough to zero.

S1.1.1 dsMTL_L21 for regression

We here specifically consider $P := 1000$ predictors, $T := 2$ tasks and $N : N_1 = N_2 = 500$ observations. Training and test data, respectively, is generated as follows. For each task $t \in \{1, 2\}$, we create a (500×1000) predictor matrix X_t with entries drawn from a standard normal distribution, as well as a (1000×1) coefficient vector W_t with entries also drawn from a standard normal distribution, aggregated in a coefficient matrix $W := (W_1, W_2) \in \mathbb{R}^{1000\times 2}$. Then, for 50% of the predictors (i.e., 50% of the rows of W), the corresponding entries are set to exactly zero for both tasks (i.e., columns of W). For each task $t \in \{1, 2\}$, a response vector $Y_t \in \mathbb{R}^{500\times 1}$ is then derived via $Y_t := X_t W_t + 0.5\delta$, where the (500×1) vector δ consists of entries drawn from a standard normal distribution.
For the MTL model calculation in both the non-private and the private cases, we here set the model hyperparameters to $\lambda := 1.2$ and $C := 1$, respectively.

S1.1.2 dsMTL_L21 for classification

We here specifically consider $P := 1000$ predictors, $T := 2$ tasks and $N : N_1 = N_2 = 500$ observations. Training and test data, respectively, is generated as follows. For each task $t \in \{1, 2\}$, we create a (500×1000) predictor matrix X_t with entries drawn from a standard normal distribution, as well as a (1000×1) coefficient vector W_t with entries also drawn from a standard normal distribution, aggregated in a coefficient matrix $W := (W_1, W_2) \in \mathbb{R}^{1000\times 2}$. Then, for 50% of the predictors (i.e., 50% of the rows of W), the corresponding entries are set to exactly zero for both tasks (i.e., columns of W). For each task $t \in \{1, 2\}$, a response vector $Y_t \in \{-1, 1\}^{500\times 1}$ is then derived via $Y_t := \text{sign}(X_t W_t + 0.5\delta)$, where the (500×1) vector δ consists of entries drawn from a standard normal distribution, and the sign function is applied component-wise.
For the MTL model calculation in both the non-private and the private cases, we here set the model hyperparameters to $\lambda := 0.01$ and $C := 1$, respectively.

S1.1.3 dsMTL_Trace for regression

We here specifically consider $P := 1000$ predictors, $T := 3$ tasks and $N : N_1 = N_2 = N_3 = 500$ observations.
Training and test data, respectively, is generated as follows. For each task $t \in \{1, 2, 3\}$, we create a (500×1000) predictor matrix X_t with entries drawn from a standard normal distribution, as well as a (1000×1) coefficient vector W_t with entries also drawn from a standard normal distribution, aggregated in a coefficient matrix $W := (W_1, W_2, W_3) \in \mathbb{R}^{1000\times 3}$. Then, we calculate the singular value decomposition of W, set the third singular value of W to zero and recompute $W = (W_1, W_2, W_3)$ based on this modification. For each task $t \in \{1, 2\}$, a response vector $Y_t \in \mathbb{R}^{500\times 1}$ is then derived via $Y_t := X_t W_t + 0.5\delta$, where the (500×1) vector δ consists of entries drawn from a standard normal distribution.
For the MTL model calculation in both the non-private and the private cases, we here set the model hyperparameters to $\lambda := 0.01$ and $C := 1$, respectively.

S1.1.4 dsMTL_Trace for classification

We here specifically consider $P := 1000$ predictors, $T := 3$ tasks and $N : N_1 = N_2 = N_3 = 500$ observations.
Training and test data, respectively, is generated as follows. For each task $t \in \{1, 2, 3\}$, we create a (500×1000) predictor matrix X_t with entries drawn from a standard normal distribution, as well as a (1000×1) coefficient vector W_t with entries also drawn from a standard normal distribution, aggregated in a coefficient matrix $W := (W_1, W_2, W_3) \in \mathbb{R}^{1000\times 3}$. Then, we calculate the singular value decomposition of W, set the third singular value of W to zero and recompute $W = (W_1, W_2, W_3)$ based on this modification. For each task $t \in \{1, 2, 3\}$, a response vector $Y_t \in \{-1, 1\}^{500\times 1}$ is then derived via $Y_t := \text{sign}(X_t W_t + 0.5\delta)$, where the (500×1) vector δ consists of entries drawn from a standard normal distribution, and the sign function is applied component-wise.
For the MTL model calculation in both the non-private and the private cases, we here set the model hyperparameters to $\lambda := 0.01$ and $C := 1$, respectively.

S1.1.5 dsMTL_Net for regression

We here specifically consider $P := 1000$ predictors, $T := 2$ tasks and $N : N_1 = N_2 = 500$ observations. To incorporate a relationship between task 1 and task 2, we specify

$$G := \begin{pmatrix} 0.5 & -0.5 \\ -0.5 & 0.5 \end{pmatrix},$$

which basically corresponds to a mean-regularized MTL, where each model is forced to approximate the mean of all models (Cao et al., 2024a,b; Evgeniou and Pontil, 2004).

Training and test data, respectively, is generated as follows. For each task $t \in \{1, 2\}$, we create a (500×1000) predictor matrix X_t with entries drawn from a standard normal distribution. Then, we generate a (1000×1) coefficient vector W_1 with entries also drawn from a standard normal distribution for task 1. Subsequently, a (1000×1) coefficient vector W_2 for task 2 is created via $W_2 := W_1 + \eta$, where η is a (500×1) vector with entries drawn from a normal distribution with mean 0 and a standard deviation in turn drawn form a continuous uniform distribution between 0 and 0.25 (with the aim to ensure a pronounced relationship between tasks 1 and 2). Then, for 50% of the predictors in task 1 (i.e., 50% of the entries of W_1) and for 30% of the predictors in task 2 (i.e., 30% of the entries of W_1), respectively, the corresponding entries are set to exactly zero. Both W_1 and W_2 are aggregated in a coefficient matrix $W := (W_1, W_2) \in \mathbb{R}^{1000 \times 2}$. For each task $t \in \{1, 2\}$, a response vector $Y_t \in \mathbb{R}^{500 \times 1}$ is then derived via $Y_t := X_t W_t + 0.5\delta$, where the (500×1) vector δ consists of entries drawn from a standard normal distribution.

For the MTL model calculation in both the non-private and the private cases, we here set the model hyperparameters to $\lambda := 0.3$ and $C := 1$, respectively.

S1.1.6 dsMTL_Net for classification

We here specifically consider $P := 1000$ predictors, $T := 2$ tasks and $N : N_1 = N_2 = 500$ observations. To incorporate a relationship between task 1 and task 2, we specify

$$G := \begin{pmatrix} 0.5 & -0.5 \\ -0.5 & 0.5 \end{pmatrix},$$

which basically corresponds to a mean-regularized MTL, where each model is forced to approximate the mean of all models (Cao et al., 2024a,b; Evgeniou and Pontil, 2004).

Training and test data, respectively, is generated as follows. For each task $t \in \{1, 2\}$, we create a (500×1000) predictor matrix X_t with entries drawn from a standard normal distribution. Then, we generate a (1000×1) coefficient vector W_1 with entries also drawn from a standard normal distribution for task 1. Subsequently, a (1000×1) coefficient vector W_2 for task 2 is created via $W_2 := W_1 + \eta$, where η is a (500×1) vector with entries drawn from a normal distribution with mean 0 and a standard deviation in turn drawn form a continuous uniform distribution between 0 and 0.25 (with the aim to ensure a pronounced relationship between tasks 1 and 2). Then, for 50% of the predictors in task 1 (i.e., 50% of the entries of W_1) and for 30% of the predictors in task 2 (i.e., 30% of the entries of W_1), respectively, the corresponding entries are set to exactly zero. Both W_1 and W_2 are aggregated in a coefficient matrix $W := (W_1, W_2) \in \mathbb{R}^{1000 \times 2}$. For each task $t \in \{1, 2\}$, a response vector $Y_t \in \{-1, 1\}^{500 \times 1}$ is then derived via $Y_t := \text{sign}(X_t W_t + 0.5\delta)$, where the (500×1) vector δ consists of

entries drawn from a standard normal distribution, and the sign function is applied component-wise. For the MTL model calculation in both the non-private and the private cases, we here set the model hyperparameters to $\lambda := 0.01$ and $C := 1$, respectively.

S1.2 Results

S1.2.1 dsMTL_L21 for regression

Regarding the example for dsMTL_L21 in case of regression (implementing a consistent selection of predictors across the two tasks), the smaller ε in the private models the more Laplace noise is added to the non-private model. Consequently, the smaller ε the more the estimated model coefficients deviate from those obtained by the non-private model (as exemplarily illustrated by the model coefficients for a specific predictor in Figure S1). For large values of ε, only a small amount of Laplace noise is injected, such that the estimated model coefficients of the respective private models strongly resemble those estimated by the non-private model.
We additionally compute the percentages of predictors wrongly selected (false positive rates, FPRs) and missed (false negative rates, FNRs), respectively, by the private models. These percentages are calculated using the sets of non-selected and selected predictors by the non-private model as corresponding reference points. There are typically far more predictors wrongly selected by the private models (Figure S2) than missed by the private models (Figure S3). While the FNR is low across all values of ε, the FPR decreases with increasing ε.

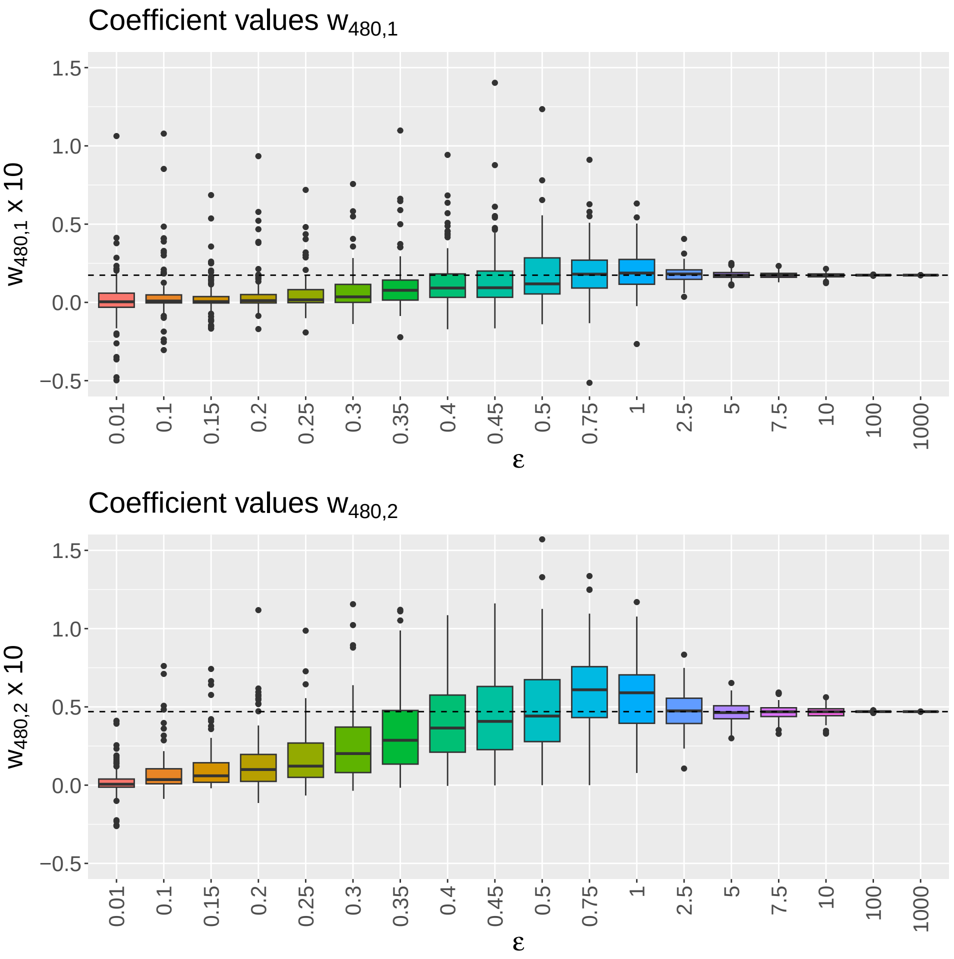

Figure S1: Differential privacy in dsMTL_L21 for regression: Boxplots relating to the distributions of model coefficients $w_{480,1}$ and $w_{480,2}$ (on a $\times 10$ scale) referring to predictor 480 (out of 1000) and tasks 1 and 2, respectively, over 100 runs, for different values of the privacy parameter $\varepsilon > 0$. For comparison, the corresponding non-private model coefficient (for $\varepsilon = \infty$) is indicated by the respective horizontal dashed line.

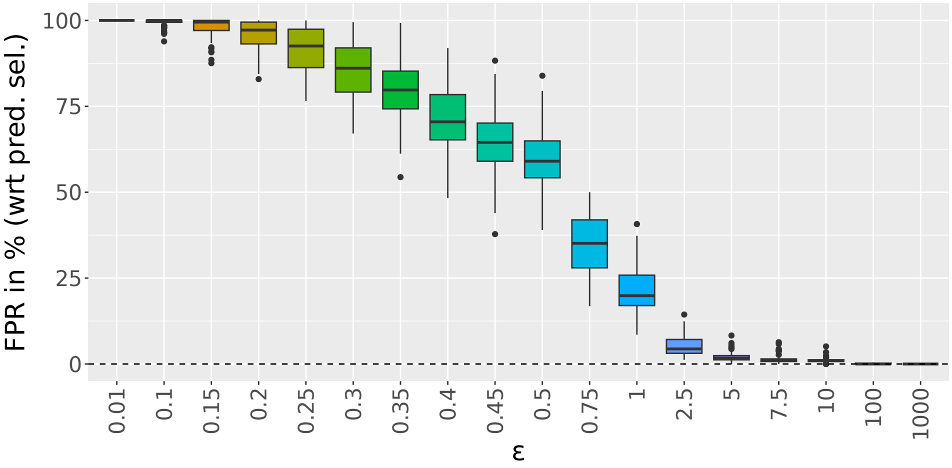

Figure S2: Differential privacy in dsMTL_L21 for regression: Boxplots relating to the distributions of the FPRs with respect to predictor selection (i.e., percentages of predictors selected by the private models, but not by the non-private model), over 100 runs, for different values of the privacy parameter $\varepsilon > 0$.

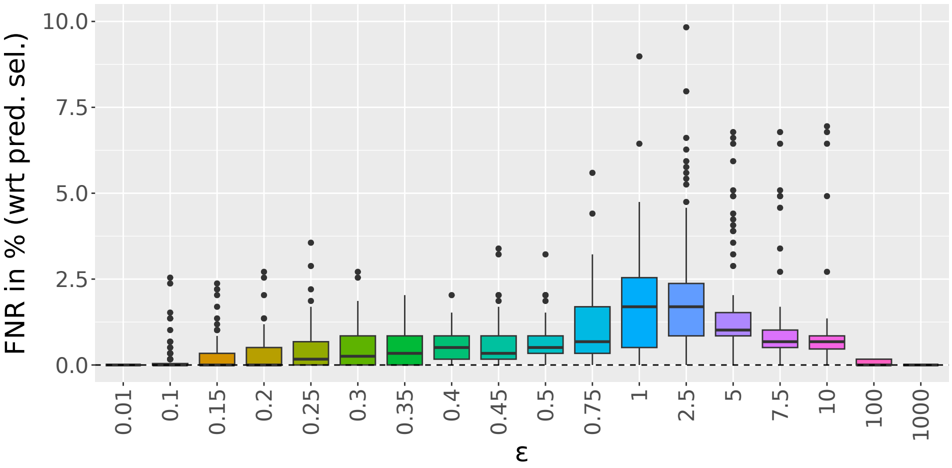

Figure S3: Differential privacy in dsMTL_L21 for regression: Boxplots relating to the distributions of the FNRs with respect to predictor selection (i.e., percentages of predictors not selected by the private models, but by the non-private model), over 100 runs, for different values of the privacy parameter $\varepsilon > 0$.

S1.2.2 dsMTL_L21 for classification

Regarding the example for dsMTL_L21 in case of classification (implementing a consistent selection of predictors across the two tasks), private models based on small values of ε clearly reduce the PL compared to the non-private model and thus are effective against MIAs (Figure S4). In contrast, private models based on large values of ε basically exhibit the same PL as the non-private model and thus fail to provide an additional protection against MIAs.

The non-private model has an average (median) AUROC of 0.740 over the two tasks. The smaller ε the more predictive skill in terms of AUROC is lost in the private models, with an unacceptable degree of performance loss for very small values of ε. In contrast, for large values of ε, the model performance from the non-private model is virtually completely retained (Figure S5).

Overall, compared to the non-private model, private models based on small values of ε offer an increased protection against MIAs, but at the cost of an immense loss of model performance. Vice versa, private models based on large values of ε virtually do not provide any additional protection against MIAs, but keep the predictive performance of the non-private model. Here, a reasonable trade-off between protection against MIAs and conservation of model performance may be realized best for private models based on values of ε in the range of around 0.2 to 0.4.

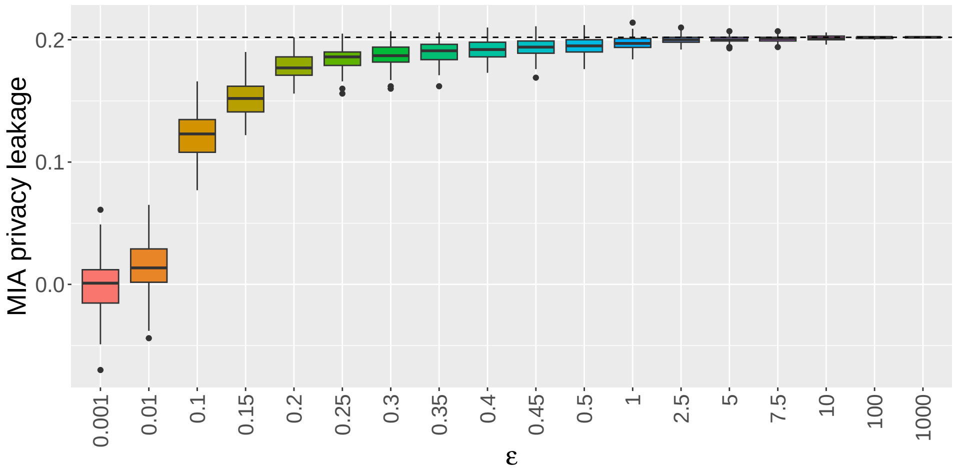

Figure S4: Differential privacy in dsMTL_L21 for classification: Boxplots relating to the distributions of privacy leakages against an MIA of the private models over 100 runs, for different values of the privacy parameter $\varepsilon > 0$. The horizontal dashed line indicates the MIA privacy leakage of the non-private model.

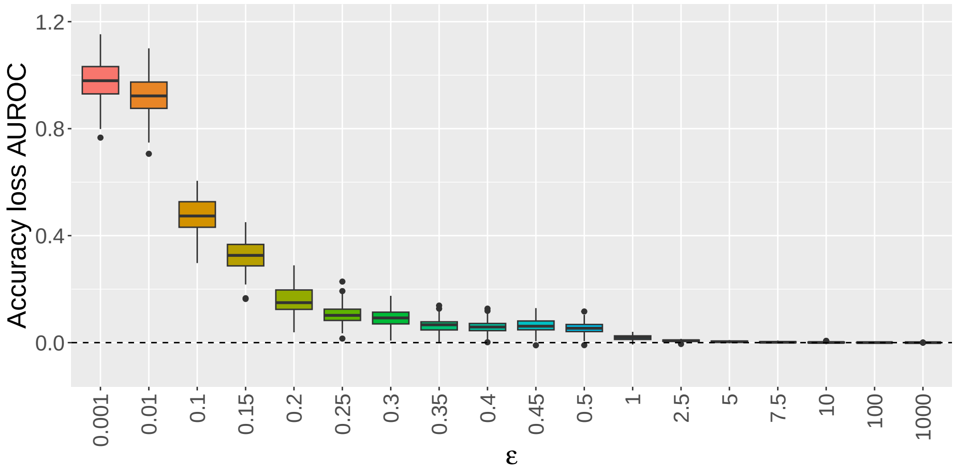

Figure S5: Differential privacy in dsMTL_L21 for classification: Boxplots relating to the distributions of accuracy losses of the private models compared to the non-private model with respect to the AUROC over 100 runs, for different values of the privacy parameter $\varepsilon > 0$. The horizontal dashed line at zero indicates an identical performance in terms of the AUROC for the non-private and the private models.

S1.2.3 dsMTL_Trace for regression

Regarding the example for dsMTL_Trace in case of regression, private models based on small values of ε clearly reduce the PL compared to the non-private model and thus are effective against MIAs (Figure S6). In contrast, private models based on large values of ε basically exhibit the same PL as the non-private model and thus fail to provide an additional protection against MIAs.
The non-private model has an average MSE of 490.89 over the three tasks. The smaller ε the more predictive skill is lost (expressed by an increase of the MSE) in the private models, with an immense degree of performance loss for very small values of ε. In contrast, for large values of ε, the model performance from the non-private model is virtually completely retained (Figure S7).
Overall, compared to the non-private model, private models based on small values of ε offer an increased protection against MIAs, but at the cost of an immense loss of model performance. Vice versa, private models based on large values of ε virtually do not provide any additional protection against MIAs, but keep the predictive performance of the non-private model. Here, a reasonable trade-off between protection against MIAs and conservation of model performance may be realized best for private models based on values of ε in the range of around 0.25 to 0.5.

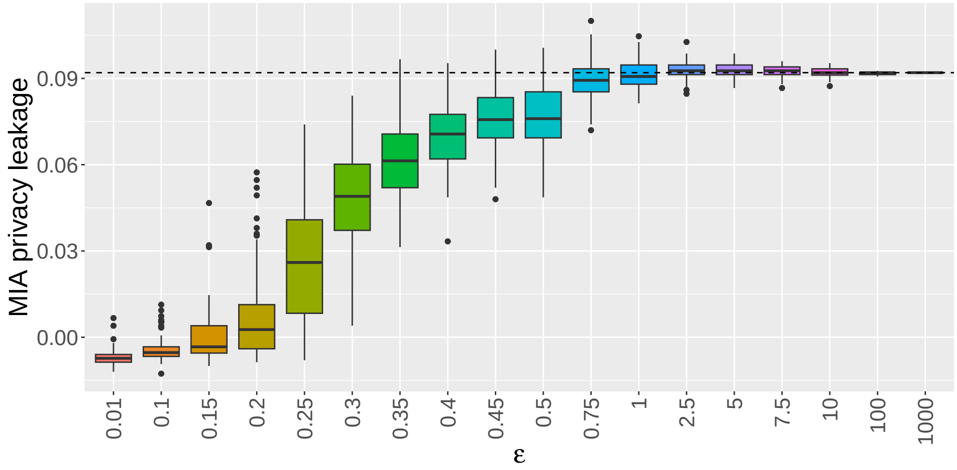

Figure S6: Differential privacy in dsMTL_Trace for regression: Boxplots relating to the distributions of privacy leakages against an MIA of the private models over 100 runs, for different values of the privacy parameter $\varepsilon > 0$. The horizontal dashed line indicates the MIA privacy leakage of the non-private model.

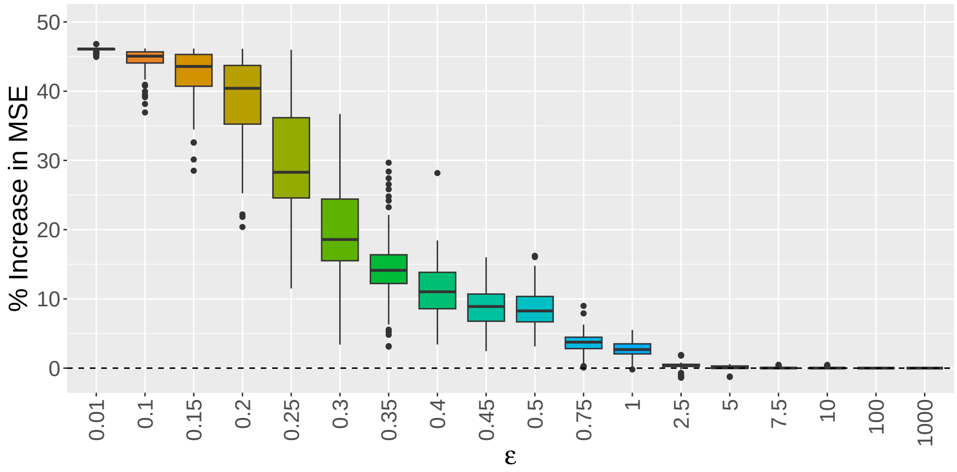

Figure S7: Differential privacy in dsMTL_Trace for regression: Boxplots relating to the distributions of percentage increases in MSE of the private models compared to the non-private model over 100 runs, for different values of the privacy parameter $\varepsilon > 0$. The horizontal dashed line at zero indicates an identical performance in terms of the MSE for the non-private and the private models.

S1.2.4 dsMTL_Trace for classification

Regarding the example for dsMTL_Trace in case of classification, private models based on small values of ε clearly reduce the PL compared to the non-private model and thus are effective against MIAs (Figure S8). In contrast, private models based on large values of ε basically exhibit the same PL as the non-private model and thus fail to provide an additional protection against MIAs.

The non-private model has an average AUROC of 0.754 over the three tasks. The smaller ε the more predictive skill in terms of AUROC is lost in the private models, with an unacceptable degree of performance loss for very small values of ε. In contrast, for large values of ε, the model performance from the non-private model is virtually completely retained (Figure S9).

Overall, compared to the non-private model, private models based on small values of ε offer an increased protection against MIAs, but at the cost of an immense loss of model performance. Vice versa, private models based on large values of ε virtually do not provide any additional protection against MIAs, but keep the predictive performance of the non-private model. Here, a reasonable trade-off between protection against MIAs and conservation of model performance may be realized best for private models based on values of ε in the range of around 0.15 to 0.25.

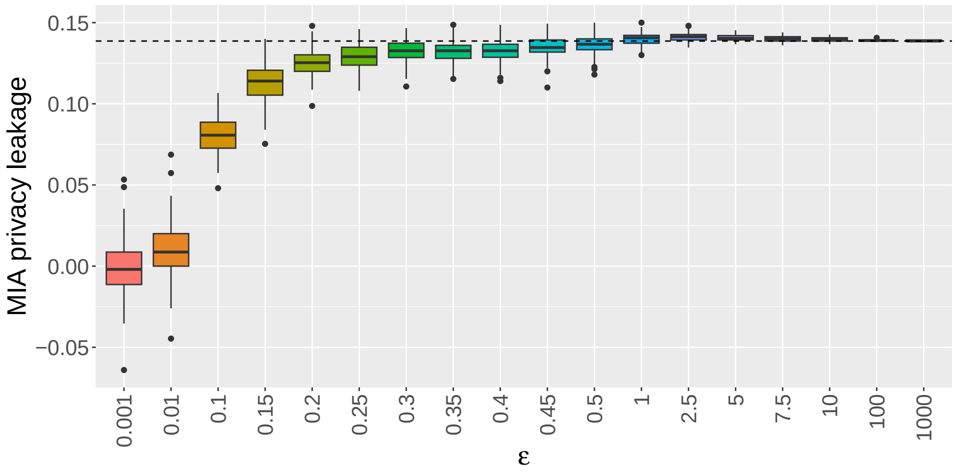

Figure S8: Differential privacy in dsMTL_Trace for classification: Boxplots relating to the distributions of privacy leakages against an MIA of the private models over 100 runs, for different values of the privacy parameter $\varepsilon > 0$. The horizontal dashed line indicates the MIA privacy leakage of the non-private model.

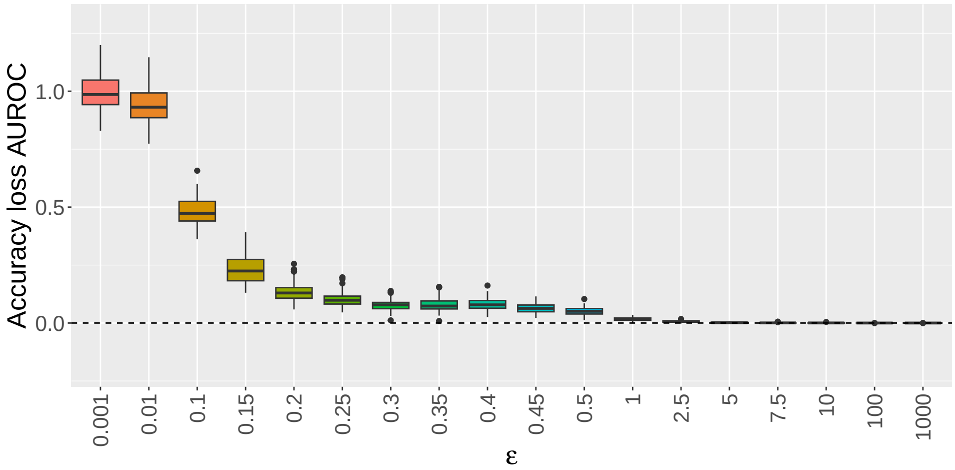

Figure S9: Differential privacy in dsMTL_Trace for classification: Boxplots relating to the distributions of accuracy losses of the private models compared to the non-private model with respect to the AUROC over 100 runs, for different values of the privacy parameter $\varepsilon > 0$. The horizontal dashed line at zero indicates an identical performance in terms of the AUROC for the non-private and the private models.

S1.2.5 dsMTL_Net for regression

Regarding the example for dsMTL_Net in case of regression (implementing a mean relationship between the two tasks, but without a consistent selection of predictors across the two tasks), private models based on small values of ε clearly reduce the PL compared to the non-private model (even attaining negative PL values) and thus are effective against MIAs (Figure S10). In contrast, private models based on large values of ε basically exhibit the same PL as the non-private model and thus fail to provide an additional protection against MIAs.

The non-private model has an average (median) MSE of 317.42 over the two tasks. The smaller ε the more predictive skill is lost in the private models (expressed by an increase in the MSE), with an unacceptable degree of performance loss for small values of ε. In contrast, for large values of ε, the model performance from the non-private model is virtually completely retained (Figure S11).

Overall, compared to the non-private model, private models based on small values of ε offer an increased protection against MIAs, but at the cost of an immense loss of model performance. Vice versa, private models based on large values of ε virtually do not provide any additional protection against MIAs, but keep the predictive performance of the non-private model. Here, a reasonable trade-off between protection against MIAs and conservation of model performance may be realized best for private models based on values of ε in the range of around 0.45 to 0.75.

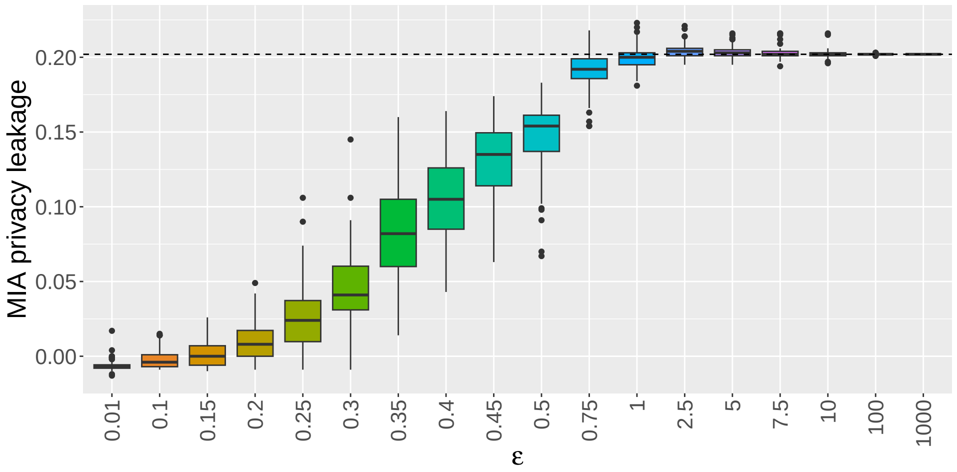

Figure S10: Differential privacy in dsMTL_Net for regression: Boxplots relating to the distributions of privacy leakages against an MIA of the private models over 100 runs, for different values of the privacy parameter $\varepsilon > 0$. The horizontal dashed line indicates the MIA privacy leakage of the non-private model.

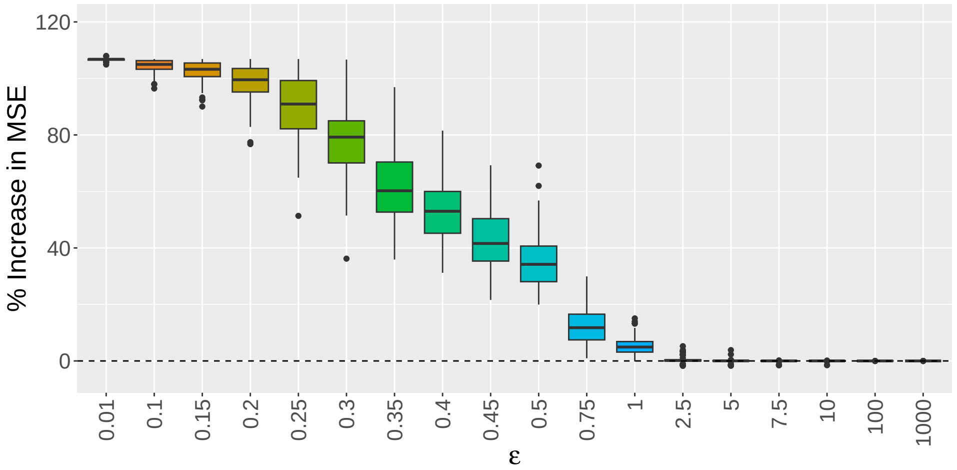

Figure S11: Differential privacy in dsMTL_Net for regression: Boxplots relating to the distributions of percentage increases in MSE of the private models compared to the non-private model over 100 runs, for different values of the privacy parameter $\varepsilon > 0$. The horizontal dashed line at zero indicates an identical performance in terms of the MSE for the non-private and the private models.

S1.2.6 dsMTL_Net for classification

Regarding the example for dsMTL_Net in case of classification (implementing a mean relationship between the two tasks, but without a consistent selection of predictors across the two tasks), private models based on small values of ε clearly reduce the PL compared to the non-private model and thus are effective against MIAs (Figure S12). In contrast, private models based on large values of ε basically exhibit the same PL as the non-private model and thus fail to provide an additional protection against MIAs.

The non-private model has an average (median) AUROC of 0.768 over the two tasks. The smaller ε the more predictive skill in terms of AUROC is lost in the private models, with an unacceptable degree of performance loss for very small values of ε. In contrast, for large values of ε, the model performance from the non-private model is virtually completely retained (Figure S13).

Overall, compared to the non-private model, private models based on small values of ε offer an increased protection against MIAs, but at the cost of an immense loss of model performance. Vice versa, private models based on large values of ε virtually do not provide any additional protection against MIAs, but keep the predictive performance of the non-private model. Here, a reasonable trade-off between protection against MIAs and conservation of model performance may be realized best for private models based on values of ε in the range of around 0.2 to 0.3.

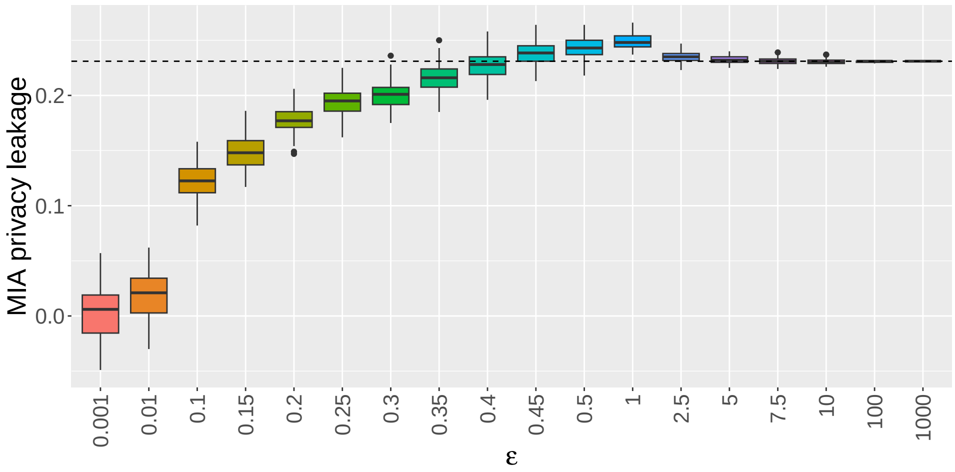

Figure S12: Differential privacy in dsMTL_Net for classification: Boxplots relating to the distributions of privacy leakages against an MIA of the private models over 100 runs, for different values of the privacy parameter $\varepsilon > 0$. The horizontal dashed line indicates the MIA privacy leakage of the non-private model.

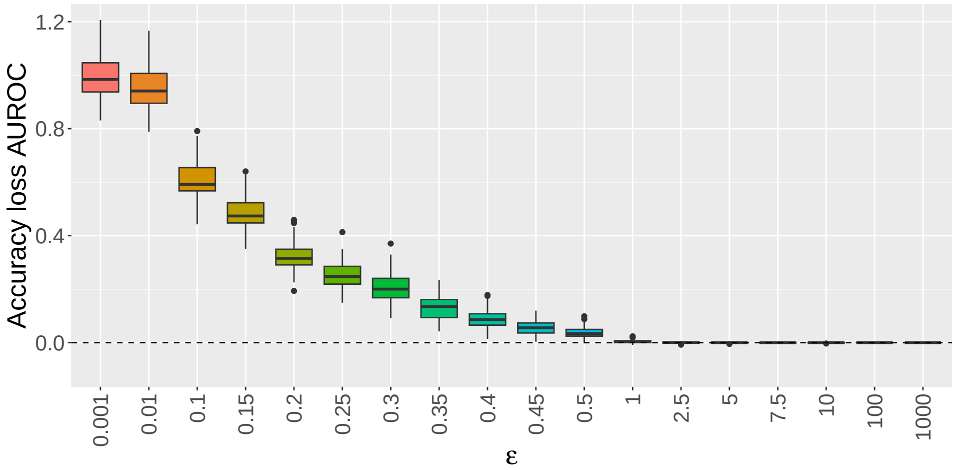

Figure S13: Differential privacy in dsMTL_Net for classification: Boxplots relating to the distributions of accuracy losses of the private models compared to the non-private model with respect to the AUROC over 100 runs, for different values of the privacy parameter $\varepsilon > 0$. The horizontal dashed line at zero indicates an identical performance in terms of the AUROC for the non-private and the private models.

S2 Extended discussion

S2.1 Non-federated versions of differential privacy implementations for MTL

While we here considered differential privacy implementations of MTL algorithms specifically in federated settings, they can also be integrated into MTL algorithms for common non-federated settings as for instance implemented in the R package `RMTL` (Cao et al., 2019). In this case, no interplay between server- and client side functions from two different R packages complementing each other via DataSHIELD is required, and the respective functions can be collected in one single R package, as usual. For the MTL variants L21, Trace and Net, which have been considered in this work here, corresponding non-federated versions are additionally bunched together in the R package `DPMTL`, which is publicly available at `https://github.com/RomanSchefzik/DPMTL`.

S2.2 Outlook and potential future work

Due to the modular nature of `dsMTL`, alternative privacy-preserving concepts, such as the less strict (ε, δ)-differential privacy, also known as approximate differential privacy (Dwork and Roth, 2014), based on Gaussian or Bernstein mechanisms, may in principle also be integrated into the `dsMTL` framework in the future.
Depending on the considered setting (i.e., the number of tasks and predictors, the chosen method etc.), computation time in `dsMTL` may get quite long, which leaves room for future improvement. Potentially, running time could be for instance reduced by integrating a suitable data compression step into the current implementation.
While we here addressed data privacy protection via a federated learning approach, an alternative notion to do so is the recently proposed Swarm Learning, "a decentralized machine-learning approach that unites edge computing, blockchain-based peer-to-peer networking and coordination while maintaining confidentiality without the need for a central coordinator, thereby going beyond federated learning" (Warnat-Herresthal et al., 2021). It is an interesting avenue for future work to investigate how MTL could be set up and performed in this context.

References

H. Cao, J. Zhou, and E. Schwarz. RMTL: an R library for multi-task learning. *Bioinformatics*, 35: 1797–1798, 2019.

H. Cao, S. Rajan, B. Hahn, M. Brenner, F. Hess, H. A. Lindner, D. Durstewitz, G. Koppe, E. Schwarz, and V. Schneider-Lindner. MTLComb: Multi-task learning combing regression and classification tasks with biomarker identification–an application to sepsis. *Journal of Critical Care*, 81:154548, 2024a.

H. Cao, S. Rajan, B. Hahn, E. Kocak, D. Durstewitz, E. Schwarz, and V. Schneider-Lindner. MTL-Comb: multi-task learning combining regression and classification tasks for joint feature selection, 2024b. URL `https://arxiv.org/abs/2405.09886`.

C. Dwork and A. Roth. The algorithmic foundations of differential privacy. *Foundations and Trends® in Theoretical Computer Science*, 9:211–407, 2014.

T. Evgeniou and M. Pontil. Regularized multi–task learning. In *Proceedings of the Tenth ACM SIGKDD International Conference on Knowledge Discovery and Data Mining*, pages 109–117, 2004.

B. I. P. Rubinstein and F. Aldà. Pain-free random differential privacy with sensitivity sampling. In *International Conference on Machine Learning*, pages 2950–2959. PMLR, 2017.

S. Warnat-Herresthal, H. Schultze, K. L. Shastry, S. Manamohan, S. Mukherjee, V. Garg, R. Sarveswara, K. Händler, P. Pickkers, N. A. Aziz, et al. Swarm learning for decentralized and confidential clinical machine learning. *Nature*, 594:265–270, 2021.
